# Calibration of High-Resolution X-Ray Tomography With Atomic Force Microscopy

**DOI:** 10.6028/jres.105.067

**Published:** 2000-12-01

**Authors:** Andrew R. Kalukin, Barry Winn, Yuxin Wang, Chris Jacobsen, Zachary H. Levine, Joseph Fu

**Affiliations:** Rensselaer Polytechnic Institute, Troy, NY 12180-3590; National Institute of Standards and Technology, Gaithersburg, MD 20899-8410; State University of New York at Stony Brook, Stony Brook, NY 11794; National Institute of Standards and Technology, Gaithersburg, MD 20899-0001

**Keywords:** atomic force microscopy, scanning electron microscope, x-ray microscopy

## Abstract

For two-dimensional x-ray imaging of thin films, the technique of scanning transmission x-ray microscopy (STXM) has achieved images with feature sizes as small as 40 nm in recent years. However, calibration of three-dimensional tomographic images that are produced with STXM data at this scale has not yet been described in the scientific literature, and the calibration procedure has novel problems that have not been encountered by x-ray tomography carried out at a larger scale. In x-ray microtomography, for example, one always has the option of using optical imaging on a section of the object to verify the x-ray projection measurements; with STXM, on the other hand, the sample features are too small to be resolved by light at optical wavelengths. This fact implies that one must rely on procedures with higher resolution, such as atomic force microscopy (AFM), for the calibration. Such procedures, however, generally depend on a highly destructive sectioning of the sample, and are difficult to interpret because they give surface information rather than depth information. In this article, a procedure for calibration is described that overcomes these limitations and achieves a calibration of an STXM tomography image with an AFM image and a scanning electron microscopy image of the same object.

A Ge star-shaped pattern was imaged at a synchrotron with a scanning transmission x-ray microscope. Nineteen high-resolution projection images of 200 × 200 pixels were tomographically reconstructed into a three-dimensional image. Features in two-dimensional images as small as 40 nm and features as small as 80 nm in the three-dimensional reconstruction were resolved. Transverse length scales based on atomic force microscopy, scanning electron microscopy, x-ray transmission and tomographic reconstruction agreed to within 10 nm. Toward the center of the sample, the pattern thickness calculated from projection images was (51 ± 15) nm vs (80 ± 52) nm for tomographic reconstruction, where the uncertainties are evaluated at the level of two standard deviations.

## 1. Introduction

X-ray tomography [1–5] is the technique of reconstructing a three-dimensional image of a sample, based on two-dimensional projection images at several viewing angles. Though a number of studies of biological sample imaging have been reported [2,3], there are few experiments on a test pattern which has been characterized by other means and imaged at the hundred-nanometer length scale [1].

Standard scientific skepticism puts the onus of calibration on the use of any new technique. For microtomography, the substantial amount of mathematical apparatus between the raw and displayed data makes the burden all the more acute. In practice, there have been many calibration studies in various fields. A few recent representative examples follow. The volume fraction of bone was obtained using computerized microtomography with the results compared to that obtained with Archimedes principle; an average underestimate of 4 % by microtomography was obtained [6]. In another study, in order to determine the rate of blood flow, the results of computerized tomography in tubes of known diameter were compared to a standard method of tracking small spheres [7]. In dentistry, computed microtomographic images of molar teeth were obtained with a resolution of 81 μm; these images were compared with physical tomography, (i.e., fine slices) and digitized video images with 25 μm resolution; a correlation coefficient *r* = 0.94 was reported [8]. In coal research, 100 μm resolution computed microtomography images were shown to compare reasonably with images obtained from color image analysis, albeit with different sensitivity to surface effects [9]. In materials science, the porosity of SiC ceramic bodies obtained with x-ray tomography was compared to optical light microscopy on physically sectioned samples [10]. Hence, researchers across many disciplines have calibrated their x-ray tomographic results using a variety of field-specific methods.

The goal of the present work is to perform a calibration of x-ray tomography on the scale length of tens of nanometers. The sample we have chosen is relatively thin, hence only the two-dimensional probes may be more appropriate for this particular sample. Nevertheless, we obtain an uncertainty estimate for scanning x-ray tomography which is applicable to more complex three-dimensional cases. The x-ray projections and tomographic images were calibrated with scanning electron microscope (SEM) and atomic force microscope (AFM) measurements of the same sample.

## 2. Experiment

**Fig. 1:**
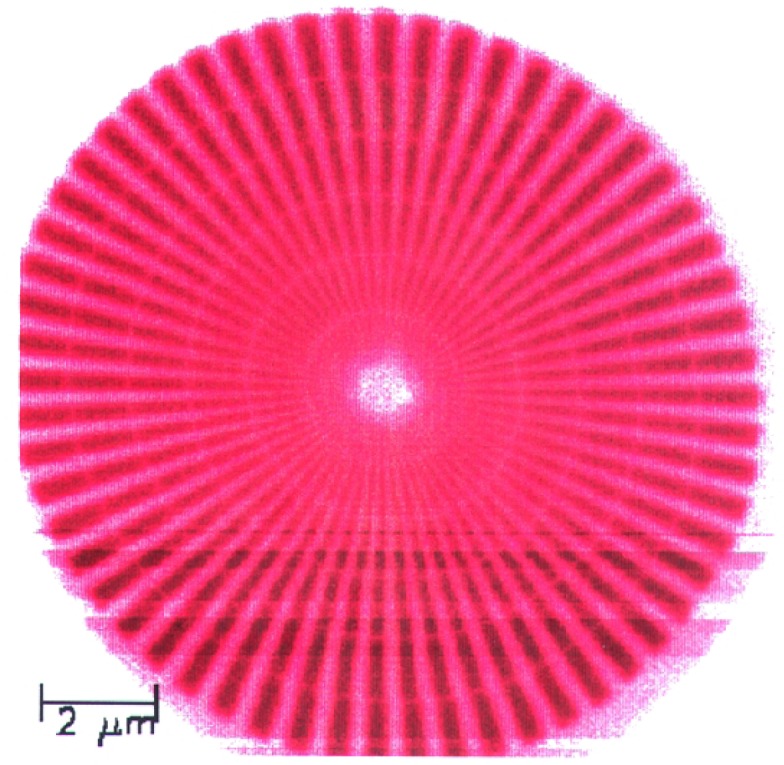
Scanning transmission x-ray microscopy image of Ge test pattern; color gray scale display. For all images in this article, brightness increases with measured thickness.

**Fig. 2:**
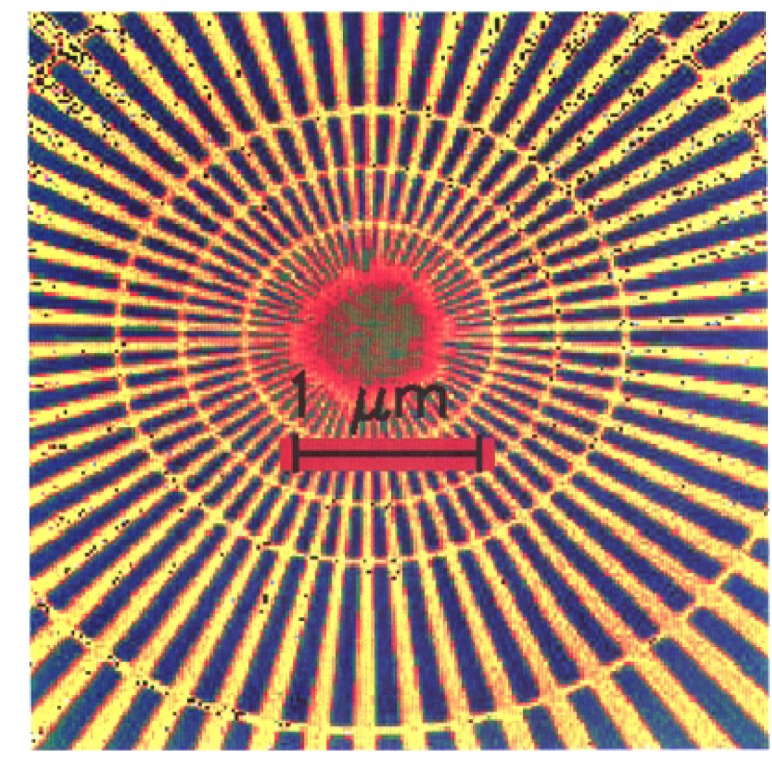
SEM image of Ge test pattern; color gray scale display.

**Fig. 3:**
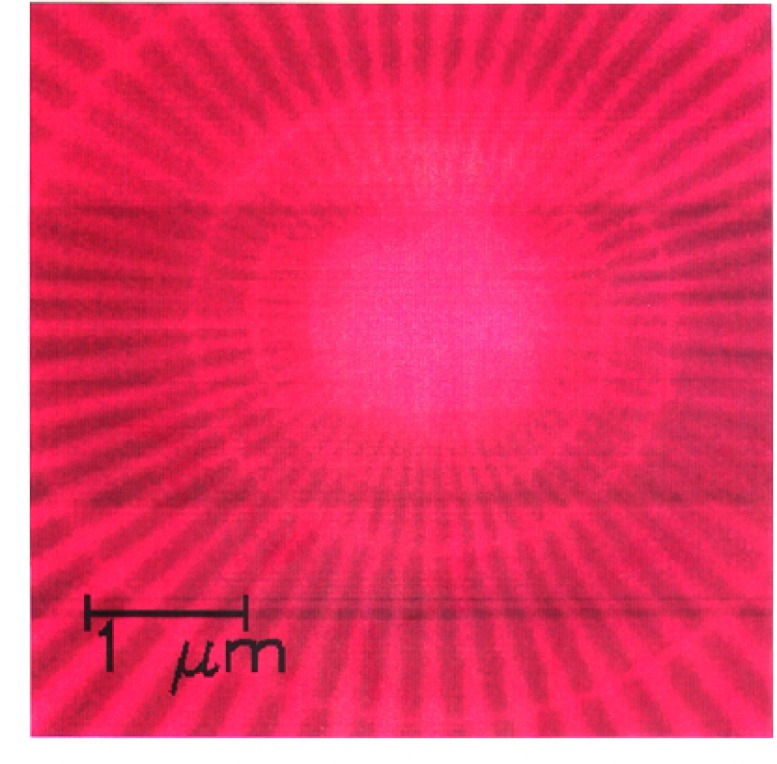
Scanning transmission x-ray microscope image of Ge test pattern; zero tilt angle, 200 × 200, 26 nm pixels; color gray scale display.

We imaged a 14.5 μm diameter star-shaped Ge pattern shown in Figs. 1 and 2, which is similar to test objects imaged earlier [2]. The concentric rings provided fiducial references for determining the line-resolution limit (i.e., minimum viewable feature size), which in the x-ray microscope image of Fig. 3 is 40 nm. The Ge spokes were deposited upon a thin silicon nitride window that was relatively transparent to x-ray beams. In addition, the sample had a 5 nm layer of Cr and a 10 nm layer of Au evenly deposited over it to prevent surface charge distortion in SEM imaging. The process used to fabricate the pattern is similar to the process described in Refs. [11] and [12]. The diameter of the entire pattern was 14.5 μm. A high-resolution AFM image (Fig. 5) reveals some contaminating particles in the center of the pattern.

The projections were acquired using the cryogenic scanning transmission x-ray microscope at the X1A beamline of the National Synchrotron Light Source, [2,4,13] using a procedure described in Refs. [5] and [11]. Coherent x rays of 585.0 eV ± 0.5 eV were focused by a 160 μm diameter Ni zone plate [12] with outermost zone width of 45 nm. For several images made at angles greater than 30°, a 160 μm-diameter Ni zone plate with outermost zone width of 60 nm was used, leading to transverse spatial resolutions of 55 nm and 73 nm, respectively. The Raleigh length (i.e., the longitudinal distance over which the transverse width of a focused Gaussian beam is within a factor of 2 of its narrowest value) of the 60 nm zone plate was 3.9 μm vs 2.2 μm for the 45 nm zone plate, which allowed the sample to remain in focus over the field of view at the larger angles. The first-order focused beam of the zone plates was selected by a 70 μm diameter order sorting aperture (OSA), combined with a 75 μm central stop on the zone plates. The sample was located at the first-order focus, 3.4 mm and 4.5 mm from the 45 nm and 60 nm zone plates, respectively. A raster scan of 200 × 200, 26 nm square pixels was made for each view. X rays transmitted through the sample were detected by a phosphor-photomultiplier system. The zone plate, pinhole, sample, and detector operated in a vacuum of 1.3 × 10^−7^ kPa, isolated from the 1.3 × 10^−10^ kPa beamline vacuum by a 100 nm thick silicon nitride window. Images were made at nineteen evenly spaced angles between −40° to +50° about the axis of rotation. A typical photon count rate of 10^−6^/s was detected in the open areas of the sample. Counts were collected for 15 ms for each pixel. The intensity of each image was normalized to regions of the sample which contained no Ge. The photon energy of 585 eV was chosen for maximum contrast between the Ge and surrounding silicon nitride window, and to match the peak of the undulator second harmonic to yield a high photon flux. The x-ray attenuation of the sample at this energy was close to the value of e^−2^ that is optimal for tomographic imaging [14].

An image of the sample at normal incidence is given in Fig. 3. The outermost complete ring in this image will be referred to by its diameter as the “3 μm ring.” The width and separation distance of each spoke along the 3 μm ring are 80 nm; for the innermost ring, these are 40 nm. Within this ring, the resolution of the zone plate we used is inadequate to distinguish the features, though the spokes do continue within the inner ring, as shown in the SEM micrograph (Fig. 2) and in images of this test pattern taken with higher resolution, shorter focal length zone plates.

In the zero-degree projection image, at the 3 μm ring in Fig. 3, the counts for the x-ray beams passing through Ge decrease to 88 % of the value for x-ray beams passing through no Ge. At the edge of the entire object the counts decrease to 59 %. This implies a thickness of 34 nm of Ge at the 3 μm ring, and 140 nm at the edge of the entire sample, based on Beer’s law and an exponential attenuation length of 265 nm [15] which is appropriate for Ge at its bulk density. However, a thin film may be deposited at a lower density. An examination of counts through the entire sample shows that the thickness of the Ge layer increases linearly outward along any radial spoke.

## 3. Image Reconstruction

**Fig. 4:**
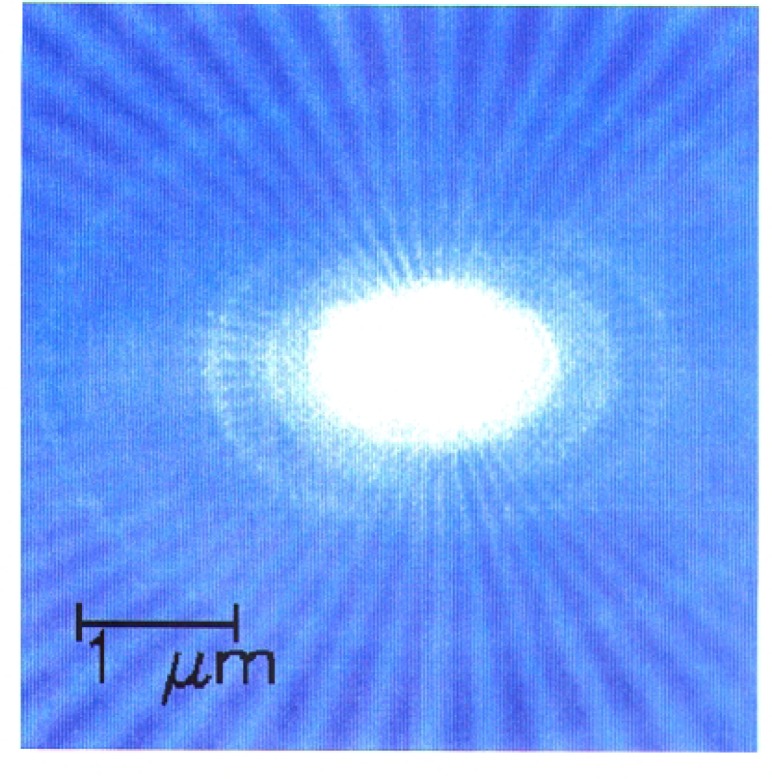
Scanning transmission x-ray microscope image of Ge test pattern; 50° tilt angle, 200 × 200 26-nm pixels; color gray scale display.

The nineteen projection images were aligned to one another in length scale and center position using the concentric rings seen in Fig. 3 as fiducial markers [16]. As the sample is turned about the horizontal axis, the rings take the shape of an ellipse (Fig. 4). In images of the rotated sample, the major axis of the ellipses should remain unchanged, and the minor axis should shrink by the cosine of the rotation angle. Distortion and angular offset errors produced discrepancies of up to 5 % in the length scales of the images, which were corrected by fitting the concentric rings in each image to an appropriate ellipse. The projections were clipped to 151 × 151 pixels, the intersection of the scanned areas after alignment.

Ideally, one would sample an object throughout a full 180° range with *N*π/2 angles, where *N* is the number of pixels in one dimension, to insure there is no loss of information [17]. Constraints of time and space required us to work with a smaller data set. Such a data set has been considered by Louis [18] whose semianalytical investigation indicates that projections taken over 90° give, in the presence of noise, define approximately one half as many basis functions as does complete angular sampling. Louis notes: “Obviously, it is well possible to reconstruct parts of the picture, namely the components connected with the singular functions belonging to the large singular values.” We performed a numerical simulation to confirm that noise-free data generated under the conditions of this experiment do indeed reconstruct a star-shaped pattern with a small, finite thickness well enough so that undersampling is a minor contributor to the uncertainties we present below.

**Fig. 5:**
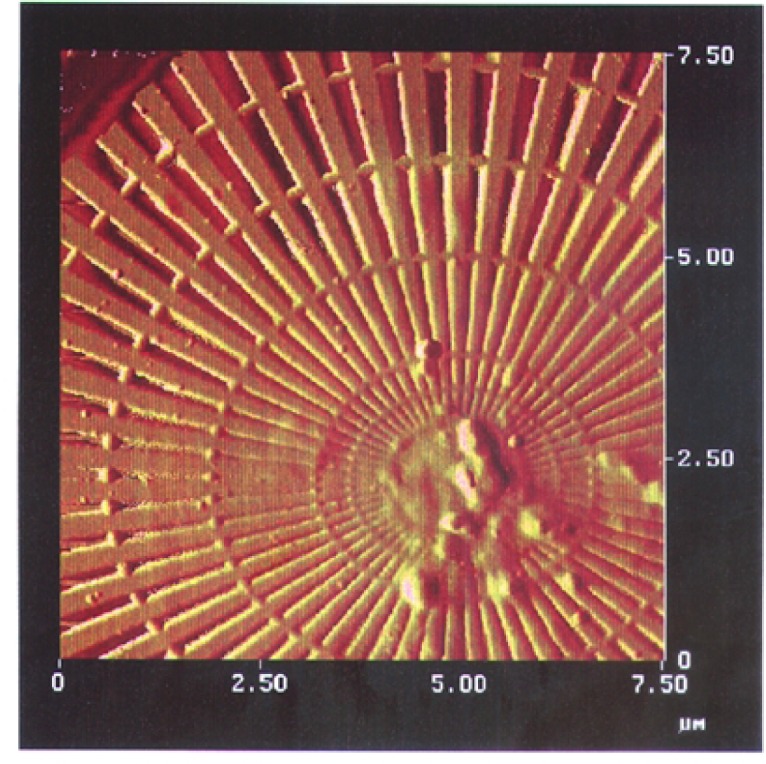
AFM image of Ge test pattern, showing contaminating particles in the inner rings; 512 × 512, 14.7 nm pixels; color gray scale display.

**Fig. 6:**
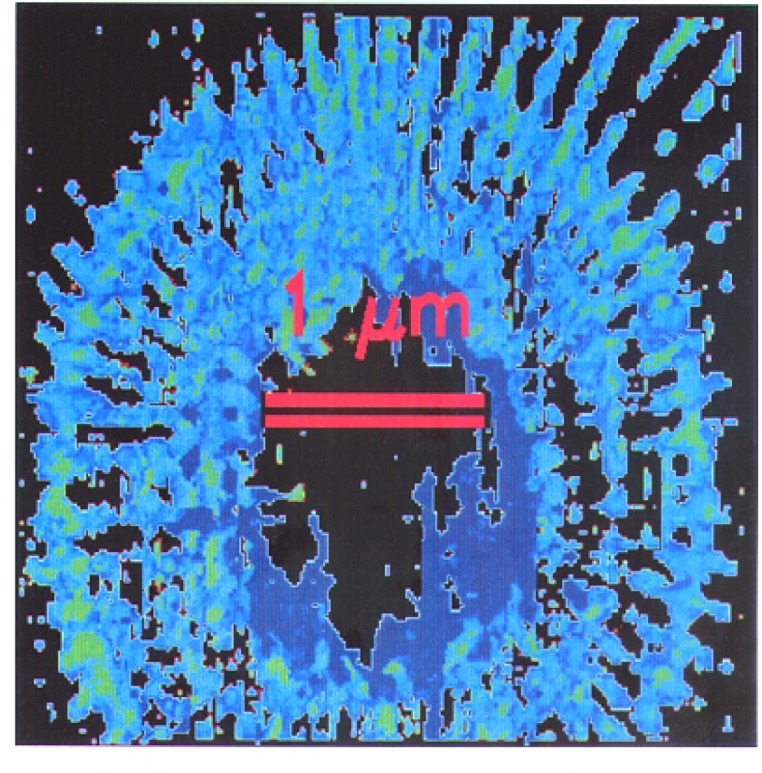
View of three-dimensional reconstruction image of test pattern, 52 nm voxels; volume isosurface display.

**Fig. 7:**
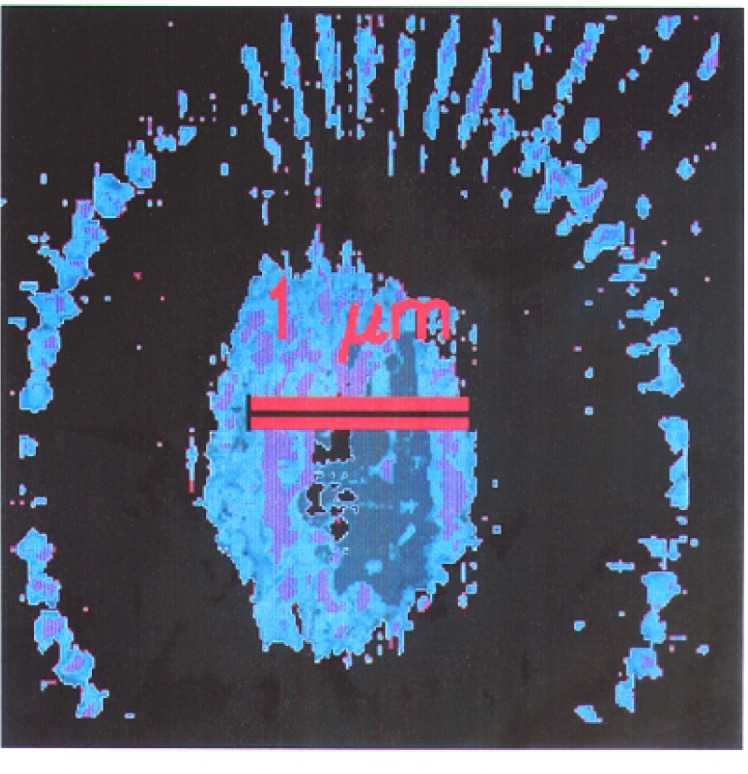
Three-dimensional reconstructed x-ray image of contamination at center of Ge test pattern, 52 nm voxels; volume isosurface display.

A three-dimensional reconstruction of the nineteen views of the sample was carried out using the Simultaneous Iterative Reconstruction Technique (SIRT) [19]. A normal incidence view of this reconstruction is shown in Fig. 6. The transverse 80 nm features are generally discernible, while smaller features closer to the center are blurred; this implies that the resolution of the three-dimensional view is degraded by a factor of two compared to the individual projection images, in which transverse 40 nm features are visible. This result is typical for the SIRT algorithm applied to experimental data. The apparent thickness of the Ge deposition in the three-dimensional object near the top or bottom of the image is (80 ± 52) nm at the thinnest parts where the uncertainty is an expanded uncertainty. The contaminating particles pictured in the AFM image in Fig. 5 are discernible from the same three-dimensional data by setting the isosurfacing of the graphical display program to show a higher thickness of material (Fig. 7).

## 4. Cross Verification

We use AFM to check the length scale given by tomography and the absorption profile. Though it would have been preferable to carry out the AFM imaging closer to the center of the sample, the resolution of the AFM would have degraded because the tip could not have reached the bottom between the spokes; furthermore, the contaminating particles near the sample center (Fig. 5) would have obstructed the AFM. However, the AFM profiles give a thickness of 210 nm at the periphery (see Table 1), while the x-ray measurements give a thickness of 140 nm, assuming the Ge film has a bulk density. This ratio is constant throughout the outward regions of the sample where the AFM measurement can be made, suggesting that the density of the Ge film is 67 % of the bulk value. In Table 1, the line “2-D X-ray scaled to AFM” is our best measurement of the thickness of the 3 μm ring, under the assumptions the the AFM measures a true height, and that the x-ray absorption measurement is a relative one due to the unknown factor of the film density.

AFM profiles of spokes at the far edge of the sample suggest that the increase in the depths of the valleys between the spokes results from the sloping sidewalls built into the structure during its construction. The sidewalls that could be measured slope by 21° to 30°, causing the spaces between the spokes within the inner five rings of the sample to be partially filled, hence the height of the spokes decreases toward the center of the pattern.

Table 1 summarizes the cross characterization of the sample by SEM, AFM, two-dimensional transmission x-ray radiography, and three-dimensional x-ray tomography. The measurements of spoke width by each technique are in good agreement. Comparison of the thickness obtained for various regions of the sample shows differences in the thickness reported by the four techniques. Tomography tends to exaggerate the thickness of objects due to imperfect alignment of the group of x-ray projections in the reconstruction program. The x-ray absorption measurement tends to underestimate the thickness of the films, because the bulk density of Ge that is used for calculation may be greater than the actual density of a thin Ge film [20].

## 5. Conclusions

We have performed a calibration of x-ray tomography on the length scale of tens of nanometers using three additional techniques: SEM, AFM, and x-ray attenuation in 2-D images. We find that these measurements are in agreement albeit with relatively large uncertainties.

## Figures and Tables

**Table 1:** Comparison of feature width and thickness measurements of Ge pattern for several imaging modalities at two regions within the pattern. All values are in nm. The assigned uncertainties are expanded uncertainties with a coverage factor of *k* = 2 (i.e., two standard deviation estimates)

Method	Bar width		Thickness	
	Star edge (nm)	3 μm ring (nm)	Star edge (nm)	3 μm ring (nm)
SEM		85 ± 10		
AFM	390 ± 60	95 ± 15	210 ± 10	> 20
2-D x-ray	390 ± 40	85 ± 40	140 ± 20	34 ± 10
2-D x-ray scaled to AFM	390 ± 40	85 ± 40	210 ± 30	51 ± 15
3-D x-ray		85 ± 52		80 ± 52
